# Genome-wide quantitative trait locus association scan of general cognitive ability using pooled DNA and 500K single nucleotide polymorphism microarrays

**DOI:** 10.1111/j.1601-183X.2007.00368.x

**Published:** 2008-06

**Authors:** L M Butcher, O S P Davis, I W Craig, R Plomin

**Affiliations:** Social, Genetic and Developmental Psychiatry Centre, Institute of Psychiatry King's College London, London, UK

**Keywords:** DNA pooling, general cognitive ability, genome-wide association, quantitative trait loci (QTLs)

## Abstract

General cognitive ability (*g*), which refers to what cognitive abilities have in common, is an important target for molecular genetic research because multivariate quantitative genetic analyses have shown that the same set of genes affects diverse cognitive abilities as well as learning disabilities. In this first autosomal genome-wide association scan of *g*, we used a two-stage quantitative trait locus (QTL) design with pooled DNA to screen more than 500 000 single nucleotide polymorphisms (SNPs) on microarrays, selecting from a sample of 7000 7-year-old children. In stage 1, we screened for allele frequency differences between groups pooled for low and high *g*. In stage 2, 47 SNPs nominated in stage 1 were tested by individually genotyping an independent sample of 3195 individuals, representative of the entire distribution of *g* scores in the full 7000 7-year-old children. Six SNPs yielded significant associations across the normal distribution of *g*, although only one SNP remained significant after a false discovery rate of 0.05 was imposed. However, none of these SNPs accounted for more than 0.4% of the variance of *g*, despite 95% power to detect associations of that size. It is likely that QTL effect sizes, even for highly heritable traits such as cognitive abilities and disabilities, are much smaller than previously assumed. Nonetheless, an aggregated ‘SNP set’ of the six SNPs correlated 0.11 (*P* < 0.00000003) with *g*. This shows that future SNP sets that will incorporate many more SNPs could be useful for predicting genetic risk and for investigating functional systems of effects from genes to brain to behavior.

The phenomenon of *general cognitive ability* was discovered more than a century ago and was called *g* to distinguish it from the many connotations of the word *intelligence*(Spearman 1904). The phenomenon is that individual differences in diverse cognitive abilities such as verbal, spatial, memory and processing speed correlate about 0.30 on average, and a general factor (an unrotated first principal component) accounts for about 40% of the total variance, as indicated in a meta-analysis of more than 300 studies (Carroll 1993; Jensen 1998). *g* is one of the most reliable, valid and stable behavioral traits, and it predicts important social outcomes such as educational and occupational levels far better than any other trait (Deary *et al.* 1994; Gottfredson 1997; Neisser *et al.* 1996; Schmidt & Hunter 2004).

The substantial heritability of *g* is documented in dozens of family, twin and adoption studies, with estimates varying from 40% to 80% and generally increasing with age (Bouchard & McGue 1981; Deary *et al.* 2006; Plomin & Spinath 2004). Most importantly, multivariate genetic analyses have consistently shown that genetic effects on cognitive abilities largely operate at the level of *g.* That is, genetic correlations among diverse cognitive abilities consistently exceed 0.50 and are often near 1.0 across diverse cognitive abilities including basic information-processing measures (Deary *et al.* 2006). This genetic overlap across cognitive abilities becomes stronger across the life span (Petrill 2002). In other words, these multivariate genetic results predict that genes found to be associated with one cognitive ability are likely to be associated with other cognitive abilities as well. Conversely, attempts to find genes for specific cognitive abilities independent of *g* are less likely to succeed, not because they do not exist (the genetic correlations are less than 1.0) but because what is in common among cognitive abilities is largely genetic and what is independent is largely environmental (Plomin & Spinath 2002). Recent multivariate genetic research has suggested that the general effects of genes on *g* extend beyond traditional cognitive abilities: genetic correlations exceeding 0.50 were also found between *g* and learning abilities and disabilities such as reading, language and mathematics (Plomin & Kovas 2005).

For these reasons, as well as for the far-reaching implications of *g* for molecular genetics (Butcher *et al.* 2006) and cognitive neuroscience (Kovas & Plomin 2006), *g* is an important target for attempts to identify genes responsible for these general effects on cognitive abilities. As for most other quantitative traits and common disorders, progress toward identifying *g* genes has been slow. A review of monogenic disorders found 282 that involve low *g*(Inlow & Restifo 2004), including phenylketonuria and fragile X syndrome, but these are rare and, in total, account for less than 1% of low *g* cases and a much smaller proportion of the normal range of variation in *g.* Dozens of common variants in candidate gene studies have been reported to be associated with *g* but, other than a small effect of the *E4* allele of *APOE*(MIM 107741) on *g* in older people (Small *et al.* 2004), no consistent replications have emerged in studies large enough (and therefore powerful enough) to detect the small genetic effects likely to underpin *g*(Payton 2006; Plomin *et al.* 2006; Posthuma & De Geus 2006; Savitz *et al.* 2006). This lack of replication is typical of candidate gene research on quantitative traits and common disorders (Hirschhorn *et al.* 2002; Ioannidis *et al.* 2001), although replication is better with larger samples (Lohmueller *et al.* 2003). The odds ratio for replicated results in candidate gene studies with large samples was only 1.2 (Ioannidis *et al.* 2006), which has daunting implications for sample sizes. For example, between 2000 and 9500 cases are required to detect a disease-conferring variant with an odds ratio of 1.4, depending on the disease allele frequency (0.50 and 0.05, respectively). Moreover, these estimates increase if the disease variant is rarer than the genotyped marker (Zondervan & Cardon 2004).

Rather than focusing on a small number of candidate genes, genome-wide linkage provides a more systematic search. From five reports of genome-wide linkage scans of *g* involving two independent samples, significant or suggestive linkage was found in three reports in a region linked in other studies to reading disability (6p25-21) (Dick *et al.* 2006; Luciano *et al.* 2006; Posthuma *et al.* 2005) but not in two other reports based on these same samples (Buyske *et al.* 2006; Wainwright *et al.* 2006). Two dozen other possible linkage regions were also reported, few of which overlapped across studies (Posthuma & De Geus 2006), which is also typical of linkage research on quantitative traits and common disorders (Altmüller *et al.* 2001). Because linkage designs are powerful for detecting genes of large effect size, one safe conclusion from these linkage studies is that there are unlikely to be any genes that have a large effect on *g*, for example accounting for more than 10% of the variance (Sham *et al.* 2000).

Allelic association is more powerful than linkage for detecting quantitative trait loci (QTLs) of small effect size (Risch 2000; Sham *et al.* 2000), but until recently, association studies have not been systematic like linkage; that is why linkage studies remained popular and association studies have been limited to candidate genes. Genome-wide association scans are now possible using single nucleotide polymorphism (SNP) microarrays (Hirschhorn & Daly 2005), although many issues remain to be resolved such as gene-centered vs. genome-centered approaches, common vs. rare variants, sample size and design (Carlson *et al.* 2004; Newton-Cheh & Hirschhorn 2005; Thomas *et al.* 2005; Wang *et al.* 2005). However, despite the decreasing costs of microarrays, they remain expensive for genotyping the very large samples needed to detect and replicate QTLs of small effect size. One economical strategy for screening large samples is to pool DNA for groups such as cases and controls for a disorder or low and high groups for a quantitative trait, which averages allele frequencies biologically for the comparison groups rather than obtaining individual genotypes and averaging them statistically (Darvasi & Soller 1994; Knight & Sham 2006; Norton *et al.* 2004; Sham *et al.* 2002). We have previously used pooled DNA in attempts to move toward more systematic association analyses of *g* in a study of 1842 short-sequence repeat markers (Plomin *et al.* 2001) and in a study of 432 nonsynonymous SNPs in genes expressed in the brain (Butcher *et al.* 2005a). However, these studies were conducted before microarrays became available and did not begin to approach genome-wide scans.

We have combined the strengths of microarrays and pooled DNA in a method we call ‘SNP Microarrays and Pooling (SNP-MaP)’. Pooled DNA can be genotyped reliably on microarrays (Butcher *et al.* 2004; Docherty *et al.* 2007; Kirov *et al.* 2006; Meaburn *et al.* 2005, 2006; Pearson *et al.* 2007). We used the SNP-MaP method with a microarray with 11 555 SNPs to identify four SNPs associated with *g* in 7-year-olds in a multistage design that included confirmation by individual genotyping of the SNPs (Butcher *et al.* 2005b). However, the average effect size of the four SNPs was just 0.2%, and these SNPs were only detected as significant because the sample was so large (*n* = 6154). Nonetheless, combining these SNPs in an aggregated *g*‘SNP set’ yielded significant associations with *g* as early as 2 years of age, significant associations with reading at 7 years and several examples of significant genotype–environment interaction and correlation (Harlaar *et al.* 2005).

Because 11 555 SNPs do not represent a genome-wide scan, the purpose of the present study was to apply the SNP-MaP approach to a genome-wide scan for *g* using the Affymetrix GeneChip® Human Mapping 500K Array set (Affymetrix, Santa Clara, CA, USA).

## Materials and methods

### Participants

The Twins Early Development Study (TEDS) is a large, longitudinal study set up to investigate the genetic and environmental bases of cognitive and behavioral development (Oliver & Plomin 2007; Trouton *et al.* 2002). The TEDS recruited families of twins born in England and Wales in 1994, 1995 and 1996. Nearly 16 000 families were contacted, of whom over 11 000 agreed to participate. Participation entailed completing booklets shortly after the birth of the twins, detailing a comprehensive range of background variables, followed by questionnaire booklets before the children's birthdays. At 7 years, 7924 children (members of 4039 twin pairs) were assessed for cognitive abilities and also provided DNA. The sample is representative of the UK population (ascertained by comparison with census data from the Office of National Statistics), although fewer mothers of twins are in full-time work outside the home. We excluded children with severe current medical problems, children who had suffered severe problems at birth or whose mothers had suffered severe problems during pregnancy. Unknown or uncertain zygosity was also grounds for exclusion as were any twins whose first language was other than English. Finally, in order to avoid issues of population stratification, we included only twins whose parents reported their ethnicity as ‘white’, which is 94% of the sample. This ‘foundation sample’, from which the children in the present study were selected, included 7089 children.

### Measures

*g* was assessed using two tests of verbal cognitive abilities and two nonverbal tests. The verbal tests consisted of the Similarities test (e.g. ‘in what way are milk and water alike?’) and the Vocabulary test (e.g. ‘what does “strenuous” mean?’), both from the Wechsler Intelligence Scale for Children (Wechsler 1992). The nonverbal tests were the Picture Completion test from the Wechsler Intelligence Scales for Children–3rd Edition UK (WISC-III-UK; Wechsler 1992) and Conceptual Grouping from the McCarthy Scales of Children's Abilities (McCarthy 1972). The tests were administered during a telephone interview, a method that has shown to be highly valid compared with in-person testing (Petrill *et al.* 2002). Prior to the telephone interview, parents were sent a sealed booklet of test items along with instructions indicating, for example, that the test booklet should not be opened prior to the telephone interview and that the twins should not be in the same room for the duration of the call.

A general factor was extracted from the four tests using principal components analysis. As the factor loadings were similar for the four tests, the *g* score used in the analysis was calculated as the sum of the standardized test scores. These unit-weighted scores correlated 0.99 with factor scores derived from the first principal component.

### Design and procedures

#### Stage 1: SNP-MaP screen of low vs. high groups

A 1 standard deviation cut-off (i.e. corresponding to the top and bottom 16th percentiles) was used to select one member of a twin pair from the *g* score distribution of foundation sample of TEDS children, resulting in 458 low *g* children and 402 high *g* children, with approximately equal numbers of boys and girls in each group. There were 653 individuals (75.9%) from this stage who were present in the pooled DNA stage of Butcher *et al.*(2005b). The mean standardized *g* score was −1.5 for the low group and 1.6 for the high group. The low and high groups were each divided into 10 independent DNA pools (biological replicates) with about 40 children in each pool; each individual was randomly assigned to one pool. The 20 DNA pools were allelotyped on Affymetrix GeneChip Human Mapping 500K Arrays as a screen for allele frequency differences between the low and high *g* groups.

#### Stage 2: testing the QTL hypothesis by individually genotyping SNPs nominated by SNP-MaP in an unselected sample

In stage 2 of the study, the QTL hypothesis was tested by individually genotyping the remaining foundation sample after excluding stage 1 individuals and selecting one twin per pair. This provided 3195 individuals representative of the entire distribution of *g* scores (*z*-score range of the sample was −3.5 to 4.9). There were 2650 individuals (82.9%) from this stage who were present in individual genotyping stage of our 10K SNP-MaP study (Butcher *et al.* 2005b). The sample provides 100%, 98% and 71% power to detect an additive single-locus genetic effect explaining 1%, 0.5% and 0.2% of the total variance of *g* scores, respectively, uncorrected for multiple testing (*P* < .05, one tailed) (Purcell *et al.* 2003).

### DNA pool construction

Genomic DNA for each individual, extracted from buccal swabs (Freeman *et al.* 2003) and suspended in Tris-ethylenediaminetetraacetic acid [EDTA] (TE) buffer (0.01 m Tris–HCl, 0.001 m EDTA, pH 8.0), was quantified in triplicate using PicoGreen™ double-stranded DNA quantification reagent (Invitrogen, Carlsbad, CA, USA). Upon obtaining reliable triplicate readings, each individual contributed the same amount of DNA to their respective pool. Because individual samples differed in their concentrations, individual DNAs were adjusted to produce equimolar DNA contributions to the pools. We deemed 1 μl the minimum volume that could be added to a pool without compromising pipette error. Therefore, the amount of DNA contributed to the pools was determined by the mass of DNA contained in 1 μl of the most concentrated individual, in this case 98.6 ng/μl. Each individual therefore contributed 98.6 ng to the DNA pool. The range of concentrations was 14.7–17.2 ng/μl for the 10 pools from the low *g* group and 15.7–17.2 ng/μl for the 10 pools from the high *g* group.

### Single nucleotide polymorphism microarray allelotyping of pooled DNA

Each of the 20 DNA pools was allelotyped using the Affymetrix GeneChip Human Mapping 500K Array set in accordance with the standard protocol for individual DNA samples (see the GeneChip Human Mapping 500K Assay manual for full protocol). Each microarray was scanned using the GeneChip® Scanner 3000 with high-resolution scanning upgrade, which was controlled using GeneChip® operating software (GCOS) version 1.4. Cell intensity (.cel) files were analyzed using gtypev.40. For quality control checks, a reference DNA individual provided by the manufacturer (sample number 100103) was also assayed on a separate microarray set.

### Generation of SNP-MaP allele frequency estimates

Relative allele signal scores, calculated using the 10K MPAM Mapping algorithm, have been shown to be reliable and valid indices of allele frequency in pooled DNA (Brohede *et al.* 2006; Butcher *et al.* 2004; Craig *et al.* 2005; Kirov *et al.* 2006; Liu *et al.* 2005; Meaburn *et al.* 2005, 2006; Simpson *et al.* 2005). We present details of how probe sets on Affymetrix GeneChip Human Mapping microarrays are used to calculate the allele frequency estimates as *Supplementary Materials and Methods* in Appendix S1. Allele frequency estimates for the 500K microarray set were calculated manually from the raw probe intensity data exported as a .txt file.

### Selection of SNPs from stage 1

To select SNPs for individual genotyping, we derived a rank-based composite score using five criteria from the stage 1 data set. The derivation of this composite score is presented as *Supplementary Materials and Methods* in Appendix S1. Briefly, the five criteria were (1) greater average allele frequency difference between low and high *g* groups, (2) smaller average variance of the low and high *g* groups (i.e. variance across the DNA pooled allele frequency estimates for each group), (3) smaller average variance within each microarray (i.e. variance across the multiple probe sets that form the microarray's allele frequency estimate), (4) greater number of successful replicate pools and (5) greater minor allele frequency, as indexed by the average of the low and high *g* groups. Because we expect many more putatively significant associations from stage 1 than could be realistically individually genotyped (>5000, *P* < .01), we used this composite to choose the top 47 SNPs with the highest composite scores. The SNP screen was restricted to the autosomes because the DNA pools included both boys and girls, which complicates analyses of SNPs on the X chromosome.

### Individual genotyping in stage 2

After excluding stage 1 individuals and selecting just one twin per pair, the 3195 individuals described earlier were genotyped using the Applied Biosystems’ SNPlex™ genotyping system and analyzed using GeneMapper version 4.0 software (Applied Biosystems, Foster City, CA, USA). SNPlex is a capillary electrophoresis-based multiplex genotyping system capable of genotyping up to 48 SNPs per sample per well (Tobler *et al.* 2005). In addition to the 3195 TEDS individuals, 88 CEPH individuals who have been genotyped as part of the HapMap Project (The International HapMap Consortium 2003, 2005) were obtained from the Coriell Institute to assess genotyping quality and error rate. For selected SNPs, reference genotypes of individuals from the Centre de l'Etude du Polymorphisme Humain (CEPH) were downloaded from HapMart, the data mining tool for downloading HapMap data (http://hapmart.hapmap.org/BioMart/martview).

Because quantitative genetic research strongly suggests that the majority of genetic effects are additive, we were primarily interested in testing SNPs for their additive effect. Therefore, genotypes of SNPs passing quality control (see below) were tested for additive genetic effects using a Pearson correlation (*r*) and coding the three observed genotypes such that 0 = AA, 1 = AB and 2 = BB. In addition, we followed a procedure recommended by Balding (2006) to test whether a nonadditive model predicted significantly better than an additive model.

### Genotyping quality control

The following sequential criteria were applied: SNPs were omitted from analysis if either poor genotype clusters prevented GeneMapper software from making calls or a SNP showed more than one genotype mismatch between CEPH genotypes deposited in HapMap and those derived using in-house genotyping methods. Individuals were omitted if their SNP call rate was <80%. Finally, for each SNP, individual genotypes were omitted if their peak heights were <25% of the average peak height for that genotypic group as measured across the entire sample.

## Results

### Stage 1: SNP-MaP screen of low vs. high groups

SNP-MaP allele frequencies for the 20 DNA pools were calculated. In order to increase the reliability of SNP-MaP allele frequency estimates, we required allele frequency estimates from a minimum of six (out of 10) replicates for both high and low groups. We also excluded SNPs with minor allele frequencies lower than 0.05 as power to detect association in this range is greatly reduced. After these exclusion criteria, the autosomal genome-wide screen consisted of 449 127 SNPs from the 500K microarray set.

**Figure 1:**
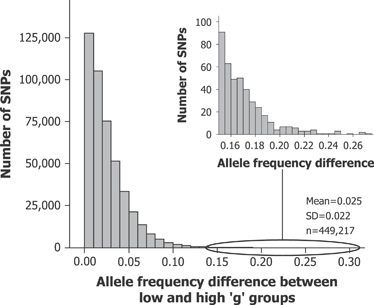
**A histogram illustrating the distribution of absolute allele frequency differences between low and high *g* groups derived through pooled DNA on microarrays.**The *y*-axis indicates the number of SNPs with differences corresponding to those on the *x*-axis. The figure shows that the vast majority of allele frequency differences are small and that the mean allele frequency between low and high *g* groups is about 0.025. The *x*-axis is elongated to accommodate outliers, which are a logical source of candidate SNPs to follow up; the extreme end of this scale is magnified for clarity and detail (inset). The total number of SNPs is less than the total number of autosomal SNPs because SNPs represented by fewer than six out of 10 replicates were removed.

The average allele frequency for the low and high *g* groups was calculated for each SNP. The correlation between the low and high *g* groups was 0.993, indicating that the rank order of allele frequencies was highly reliable overall – a test analogous to genome control. Accordingly, between-group differences were small: Figure 1 illustrates that 90% of the SNPs exhibited between-group differences smaller than 0.05, with a mean between-group absolute difference of 0.025 for the whole data set (range: 0.00–0.27).

**Figure 2:**
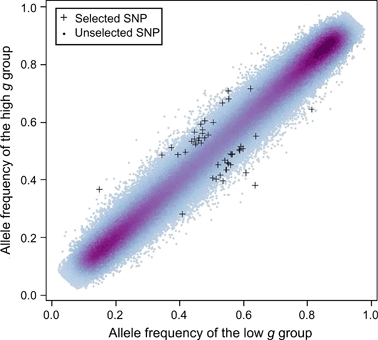
**A scatter plot showing the 47 top-ranked SNPs (crosses) against the background of unselected SNPs comparing allele frequencies for the low *g* group (*x*-axis) and the high *g* group (*y*-axis).**The figure also displays the density of SNPs as a function of low *g* vs. high *g* allele frequency differences; density of SNP clusters increases as the heat map changes from light (sparse clusters) through to dark (dense clusters). Allele frequency differences are small, with the majority of small differences occurring for SNPs with minor allele frequencies of 0.10–0.25, which reflects the overrepresentation of SNPs with these allele frequencies on the Affymetrix microarray. The correlation between low and high *g* allele frequencies was 0.993, indicating high reliability across the two groups.

As explained in *Materials and methods*, SNPs selected for individual genotyping were chosen on the basis of a ranked composite score, which took into account the between-group allele frequency difference, variance between and within biological replicate microarrays, number of successfully assayed arrays and minor allele frequency. Because of financial restrictions, we were limited to genotyping a single probe set of 47 SNPs with the highest composite scores. The mean absolute difference between low and high SNP-MaP allele frequency estimates for these was 0.108 (ranging from 0.05 to 0.26). The SNP with the largest difference was not selected as it exhibited high levels of variance and the minimum number of replicates, which counted unfavorably in the composite measure. Figure 2 illustrates the 47 selected SNPs in the context of the full data set by plotting the average allele frequency of the low *g* group against that of the high *g* group. Using conventional parametric statistical methods (Student's *t*-test) to test for allele frequency differences between the 10 pools for the low *g* group and the 10 pools for the high *g* group, all SNPs were significant at *P* < 0.05 (range: 0.0000002 ≤ *P*≤ 0.03). However, the composite selection criterion goes beyond traditional *P*-values to incorporate variance between and within groups, as well as the variance of a single SNP's measurement on a single microarray. Details about the 47 selected and successfully genotyped SNPs can be found in Table S1 in Appendix S1.

### Individual genotyping quality control

On a study-wide level of analysis, five out of 47 SNPs failed to call because of poor genotype clustering and were omitted; five SNPs were also omitted that showed unacceptably high genotyping error rates (measured by the concordance between our in-house-derived genotypes for 88 CEPH individuals and the genotypes of the same CEPH individuals available from HapMap); for the 37 SNPs passing these quality control criteria, we observed 9 mismatches out of 2981 genotypes (error rate 0.3%). Of these errors, there was bias toward calling heterozygotes.

Finally, 13% of the sample showing unacceptably low call rates (<80%) were omitted; fragmented DNA is a prerequisite to running SNPlex, and overfragmentation is the likely cause of these low call rates. At the SNP level of analysis, we excluded an additional approximately 4% of SNP genotypes whose peak height was <25% of the average peak height for that SNP across the study. At the cost of reduced sample size, these conservative criteria improved observed genotypic distributions under Hardy–Weinberg expectation, tightened genotype clusters and left the distribution of *g* unchanged. The effective sample size was *n* = 2782.

### Stage 2: testing the QTL hypothesis by individually genotyping SNPs nominated by SNP-MaP in an unselected sample

The 37 successfully genotyped SNPs nominated by stage 1 were individually genotyped across the unselected sample of 2782 children in order to test the QTL hypothesis directly by assessing the extent to which the SNPs are associated with *g* throughout the distribution. Each individual's genotypes for the 37 SNPs were tested for additive genotypic effects. With 37 tests and an alpha of 0.05, two significant results would be expected on the basis of chance alone. As shown in Table 1, six SNPs (16%) were significantly associated with individual differences in *g* throughout the distribution using a nominal one-tailed alpha level of 0.05 (range: 0.0007 ≤ *P*≤ 0.043). We used a one-tailed test because the difference observed in stage 2 was required to be in the same direction as that seen in stage 1 screening. None of the six SNPs significantly deviated from Hardy–Weinberg expectation. A summary of stage 1 and 2 results for all 37 SNPs (including SNP locations) is provided in Table S1. After correcting for 37 simultaneous tests using a step-up false discovery rate (Benjamini & Hochberg 1995) of 0.05, only the most strongly associated SNP remained significant (corrected *P* < 0.03). However, the other nominally significant SNPs were also included in our SNP set (see below).

**Table 1:** Mean quantitative trait scores and correlations between additive genotypic values and quantitative trait scores

SNP ID	Chromosome	Genomic location	Genotype	*n*	Mean *g* score (SD)	*r*	Estimated effect size (%)
rs11691504	2q31.3	Intergenic	AA*	601	−0.07 (1.03)	0.042 (*P* = 0.015)	0.2
			AC	1368	0.03 (0.98)		
			CC	711	0.05 (0.98)		
rs1378810	3q22.1	Intron 55 (DNAJC13)	AA*	515	−0.06 (0.98)	0.062 (*P* = 0.0007)	0.4
			AT	1362	−0.02 (1.00)		
			TT	790	0.11 (0.99)		
rs2496143	6p24.1	Intron 5 (TBC1D7)	CC	1005	0.06 (1.00)	−0.034 (*P* = 0.037)	0.1
			CT	1321	−0.01 (1.01)		
			TT*	378	−0.04 (0.92)		
rs11761076	7q32.1	Intergenic	AA	447	0.10 (0.94)	−0.045 (*P* = 0.010)	0.2
			AG	1316	0.03 (1.01)		
			GG*	881	−0.04 (1.00)		
rs174455	11q12.3	Intron 1 (FADS3)	AA	1085	−0.02 (0.94)	0.043 (*P* = 0.013)	0.2
			AG	1276	0.02 (1.02)		
			GG*	329	0.12 (1.03)		
rs7195954	16p13.3	Intergenic	CC	253	−0.01 (0.99)	0.033 (*P* = 0.043)	0.1
			CG	1128	−0.02 (1.01)		
			GG	1274	0.06 (0.97)		

Because additive genotypic coding was initially alphabetically ordered (0 = AA, 1 = AB and 2 = BB), the resulting codes produce negative correlations for SNPs rs11761076 and rs2496143, indicating lower *g* scores for the B alleles of these SNPs. By squaring the Pearson correlation coefficient (*r*), an effect size of the SNP on *g* can be estimated. The ancestral allele of SNP rs7195954 was not known at the time of writing.

* Individual homozygote for the ancestral alleles of the SNP.

The significant correlations are small, with an average correlation (*r*) across the six SNPs of 0.043; the largest correlation is 0.062 (rs1378810, *P* = 0.0007). Squaring these correlations (*r*^2^) to estimate effect size indicates that these associations account from only 0.1% to 0.4% of the variance of *g* scores. The average effect size is 0.2%, and the sum of the effect sizes of the six SNPs is 1.2%. Following the procedure suggested by Balding (2006), we compared additive and nonadditive models and found that the additive model fits best for all six SNPs (data not shown). We also examined the associations separately for boys and girls, but no significant differences were found; because our screening design included boys and girls, it would favor SNPs that show effects in both sexes.

**Figure 3:**
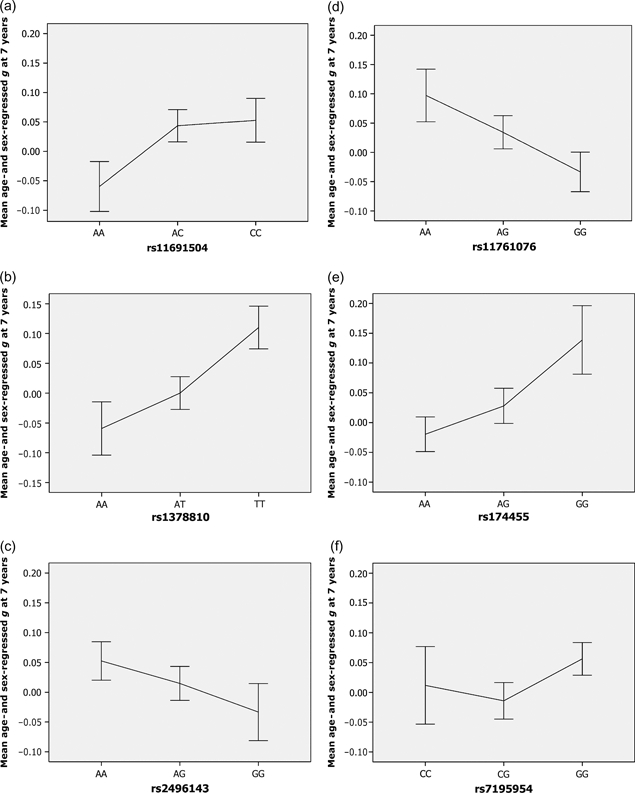
**Genotype-by-phenotype plots illustrating the effect of genotype (*x*-axis) on standardized *g* scores (*y*-axis).**The six significantly associated SNPs using individuals genotyping are labeled a–e.

Figure 3 presents the results for the six significant SNPs in terms of standardized mean quantitative trait *g* scores (age and sex regressed) for the three SNP genotypes. For the first five SNPs, the nonoverlapping standard error bars indicate that the homozygote genotypes differ significantly on *g*. For the sixth SNP (Fig. 3f), the significant difference is between the heterozygote (CG) and one homozygote (GG), the genotypes with the largest sample sizes. Because the *g* score is standardized, it provides another way to consider the effect sizes of these associations by comparing the *z*-score differences between the homozygote genotypes. For example, the average *z*-score difference between the homozygotes for the six SNPs is 0.12 (range: 0.07–0.17), indicating that the homozygotes differ by about 0.12 of a standard deviation in their *g* scores (approximately 2 IQ points on a scale with a mean of 100 and a standard deviation of 15). This finding indicates that even with very modest correlations such as these, homozygotes that differ at a single locus could provide biological clues into understanding the etiology of *g*. This point takes on greater significance when the SNPs are combined in an SNP set.

### SNP set

The additive genotypic values for the six SNPs are uncorrelated because the SNPs are not in linkage disequilibrium with each other. This permits the creation of a composite SNP set that aggregates the small effects of each SNP and can be useful for certain purposes such as selecting individuals at genotypic risk in studies that are not sufficiently large to provide the power needed to analyze each SNP separately. Single nucleotide polymorphism genotypic values were recoded in a reversed direction for SNPs rs2496143 and rs11761076 so that high genotypic values for all SNPs indicate higher *g*.

**Figure 4:**
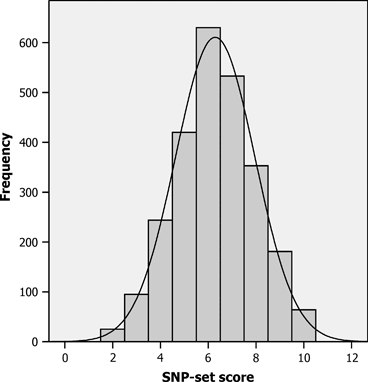
**A histogram illustrating the distribution of SNP-set scores.**The *x*-axis scale runs from 0 to 12 because each of the genotypes for the six SNPs is coded using an additive model with 0, 1 or 2 ‘increaser’ alleles. For each SNP, scores of 2 indicate that the individual is homozygous for the allele conferring higher *g* scores. These scores are summed at each locus for each individual to create an SNP-set score. The *y*-axis indicates the number of individuals with a particular SNP-set score. The majority of individuals score between 5 and 8 because the SNPs were chosen on the basis of high minor allele frequency and thus high heterozygosity.

In theory, summing the SNP genotypes for the six significant associations can produce SNP-set scores from 0 to 12. In practice, however, the low combined probability that individuals will be homozygous for either all low or all high alleles results in SNP-set scores that ranged from 1 to 11, with only three individuals with a SNP-set score of 1 and only nine individuals with a SNP-set score of 11, which is close to the expectation of 3 and 10, respectively, based on the observed genotypic frequencies. Complete genotype data for all six SNPs were available for 2557 individuals. An additional 119 individuals who had a minimum of four genotypes of the six SNPs in stage 2 were included in this analysis (*n* = 2676) using a missing data option that substituted the population mean genotypic value for missing SNPs. SNP-set scores were normally distributed (Fig. 4).

**Figure 5:**
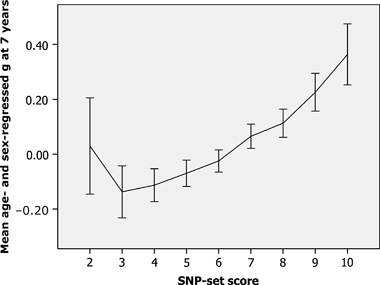
**A genotype-by-phenotype plot illustrating the relationship between SNP-set scores and standardized *g*.**The correlation between the SNP-set scores and the *g* scores is 0.105 (*P* < 0.00000003, *n* = 2676). The association is approximately linear, which indicates additivity of the genotypic values in the SNP set.

The correlation between SNP-set scores and *g* scores is 0.105 (*P* < 0.00000003, *n* = 2676). Squaring this correlation indicates an effect size of 1.1% that is comparable to the sum of the effect sizes of the six SNPs, which, as noted earlier, was 1.2%. The correlation between SNP-set scores and *g* scores was essentially unchanged when the individuals with missing data were excluded (*r* = 0.100, *P* < 0.0000002, *n* = 2557). Figure 5 plots the standardized *g* score against the SNP-set scores for the sample of 2676 individuals. It can be seen that the association is approximately linear, which indicates additivity of the genotypic values in the SNP set. The standardized *g* score difference between SNP-set scores below 3 and above 8 is 0.24 SD, comparable to a difference of about 4 IQ points. This difference suggests that selecting on the basis of extreme SNP-set scores could be effective for selecting groups with a genetic liability for low or high *g*, although the associations of the individual SNPs yield small effect sizes.

## Discussion

In this first genome-wide association scan employing approximately 500 000 SNPs for general cognitive ability (*g*), six SNPs survived our two-stage screen for QTLs that are associated with *g* across the normal distribution. In stage 1, which compared allele frequencies derived from pooled DNA for low and high *g* groups, a composite of five criteria was used to nominate SNPs for individual genotyping in stage 2. In stage 2, for financial reasons, a single SNPlex multiplex of 47 SNPs was used to individually genotype an independent and representative sample of 3195 individuals. Using an alpha of 0.05 to provide a reasonable balance between false positives and false negatives in the search for QTLs of very small effect size, six SNPs yielded significant associations, whereas only two significant associations would be expected on the basis of chance.

### Small effect sizes and the use of SNP sets

Genome-wide association is a powerful and systematic tool for identifying QTL associations; however, a finding of general significance is that no associations greater than 0.5% were detected although the effective sample of >2500 provided 95% power to detect them. Given the increased SNP density of the Affymetrix 500K microarray, it is surprising that this result does not substantially improve upon our previous work in cognitive and learning abilities and disabilities using unmultiplexed assays as well as 10K and 100K microarrays (Butcher *et al.* 2005a,b; Meaburn *et al.* 2007). One explanation is that the sheer number of SNPs exacerbates the problem of detecting weaker – but true – signals from the noise caused by 500 000 SNPs. For example, although the expected proportion of true-positive associations should be larger in a panel of 500 000 SNPs than, say, 100 000 SNPs, the number of false positives should be proportionately even greater. This problem may have contributed to the fact that our previous study of *g* using 10K SNP microarrays identified four SNPs (Butcher *et al.* 2005b), whereas the present study with 50 times more SNPs identified only six SNPs. Moreover, although we had expected that the 500K SNP microarrays would identify a few SNPs with larger effect sizes, the average effect sizes of associations in the two studies were the same. Because none of the four SNPs from our previous study is on the Affymetrix 500K microarray set, we cannot directly compare SNP-MaP results for the two studies. From an indirect perspective, there was only one perfect proxy on the Affymetrix 500K microarray for any of the SNPs from our previous study (Butcher *et al.* 2005b). According to HapMap (The International HapMap Consortium 2005) data for the CEPH population, only one SNP (rs11385352, from the 500K microarray) perfectly tags (*r*^2^ = 1) a SNP (rs991684, from the 10K microarray) reported in our previous study; yet, it showed no association in the current study. Association may, however, have been masked by the between-group variance for rs11385352.

One possible reason for not observing larger, common, single-locus SNP effects for *g* is that they do not exist. Genome-wide association scans based on large samples can identify SNPs with large effect sizes, as shown in research on macular degeneration (Klein *et al.* 2005) and inflammatory bowel disease (Duerr *et al.* 2006). By itself, this possibility warrants the use of genome-wide association scans. However, it may be that for common disorders and quantitative traits such as the present genome-wide scan for *g*, the main finding is the *exclusion* of SNPs of large effect size to the extent that coverage for common variants is virtually complete. If the largest SNP effects are as small as 0.4% of the variance, winnowing the wheat from the chaff will be difficult, requiring extremely large samples, multiple-stage designs and replication in independent samples. Alternatively, it may be that there are few common SNPs of large effect and that there are many more rare variants of larger effect size. Our data cannot address this issue because, for reasons of power, we selectively chose SNPs whose minor allele was common. However, several recent genome-wide association studies of common disorders including obesity, heart disease, type 2 diabetes and bipolar disorder also only found associations of very small effect size (Diabetes Genetics Initiative of Broad Institute of Harvard and MIT, Lund University, and Novartis Institutes for BioMedical Research *et al.* 2007; Wellcome Trust Case Control Consortium 2007). Moreover, the authors of these studies acknowledge the ‘winner's curse’– the phenomenon whereby an initial study overestimates the genetic effect size. This suggests that initial discoveries of associations are liberal estimates of effect size in that they capitalize on chance, and that the key to replicable SNP associations is even larger samples. Nonetheless, the substantial heritability of most common disorders and quantitative traits such as *g* means that DNA polymorphisms are associated with the disorders and traits, and we must do what it takes to find the genes responsible for this genetic influence.

Until the six SNPs reported in this study are replicated in other samples, caution is in order because their average effect size is only 0.2% of the variance of *g* in the representative unselected sample used in stage 2. The small effect sizes will make replication difficult because a sample of about 4000 individuals is needed to reach 80% power (*P* < 0.05, one tailed) to detect an effect size of 0.2%. However, the composite SNP set could be tested for replication in much smaller samples. The SNP set of five SNPs has an effect size of about 1% of the total variance of *g*. An effect size of 1% would require a sample of about 780 to reach 80% power to detect an association between SNP set and *g*.

### SNP locations

Although none of the six SNPs are functionally related to *g*, the genomic features surrounding the locations of the six SNPs are interesting. Three of the SNPs (rs1378810, rs2496143 and rs174455) are in known genes [DnaJ (Hsp40) homologue, subfamily C, member 13 (*DNAJC13*), TBC1 domain family, member 7 (*TBC1D7*) and fatty acid desaturase 3 (*FADS3*), respectively], but none are in coding regions; given that only 1.7% of the SNPs on the Affymetrix 500K microarray are in coding regions [Affymetrix annotation data (July 2007)], this is hardly surprising. One SNP (rs7195954) lies in a region of known copy number variation (CNV) and may warrant more detailed CNV analysis. One SNP (rs11761076) is in close proximity (27 kb) with a gene but shows no evidence of being in linkage disequilibrium (LD) with it. One SNP (rs11691504) lies in large gene desert with no currently documented functional elements nearby. Finally, and potentially most interesting, is the high LD between rs1378810 and the 3′ untranslated region (UTR) of *DNAJC13*, which contains nine predicted conserved mammalian micro RNA (miRNA) regulatory target sites. None of the SNPs show a high degree of sequence conservation with other species, which may indicate human-specific effects.

### Limitations

One important limitation is that only 47 of the SNPs nominated by SNP-MaP in stage 1 were individually genotyped and tested for replication in the independent stage 2. In the future, we plan to mine these SNP-MaP data further. However, rather than enduring the expense of individually genotyping large numbers of SNPs, our ongoing research has converted stage 2 to a second independent SNP-MaP screen comparable to the stage 1 SNP-MaP design with 10 independent DNA pools created from a low *g* group and 10 pools from a high *g* group. By allelotyping these 20 DNA pools on 500K SNP microarrays, we can conduct much more thorough stage 2 screening. A representative sample will be individually genotyped in stage 3 to test for SNPs that pass the hurdles of stages 1 and 2.

Another limitation is that more sophisticated ways of analyzing pooled DNA are now available, for example ‘GenePool’ (Pearson *et al.* 2007), as well as increased literature dedicated to design considerations, for example Macgregor's frameworks for minimizing measurement variance (Macgregor 2007). However, these methods were not available when we completed our SNP-MaP screen and selected SNPs for individual genotyping. Nonetheless, our multiple-criteria composite for selection that incorporates variance across independent DNA pools is central to these newer analytic strategies.

Our study may potentially be limited in six other ways:

1. The SNP-MaP screen did not use *k*-corrected allele frequency estimates. *k*- correction improves the accuracy of absolute estimates of allele frequency for pooled DNA (Simpson *et al.* 2005). However, *k*-correction does not have much effect on *relative* estimates of allele frequency differences, which is the relevant issue when comparing pooled DNA for groups (Meaburn *et al.* 2006). Because *k*-correction is SNP specific, it is difficult to speculate on which SNPs would have been chosen had *k*-correction data been available.
2. SNPs with minor allele frequencies less than 5% were excluded in the SNP-MaP screen for reasons explained earlier. Moreover, our multiple-criteria composite preferentially selected SNPs with high minor allele frequencies (Fig. 2). It seems reasonable to begin by testing the common variant/common disease QTL hypothesis (Cargill & Daley 2000); in the future, we could investigate less common alleles (in which case, *k*-correction becomes more appropriate).
3. We did not observe SNP effects accounting for greater than 0.4% of the variance; however, this does not necessarily mean that single-SNP effects accounting for more of the variance on *g* do not exist. The power to detect effects is also contingent on the sensitivity of the SNP-MaP approach as well as the coverage of the 500K microarray (66% of common variants captured at *r*^2^ > 0.80 using a single-marker approach; Pe’er *et al.* 2006), which are two areas likely to improve in time.
4. The SNP-MaP screening did not discriminate SNPs in known regions of copy number variants (Redon *et al.* 2006; Wong *et al.* 2007). The Affymetrix 500K microarray that we used is not ideally suited to detect copy number variants; however, the Affymetrix 5.0 (including 420K nonpolymorphic probes) and Affymetrix 6.0 (including 946K nonpolymorphic probes) microarrays are much better positioned to detect and identify copy number variants. However, if copy number variants were important, we suggest that they would add noise to our current SNP-MaP screening, thus increasing false negatives rather than false positives.
5. Our sample was 7 years old and results might differ at other ages. Although quantitative genetic research indicates that genetics largely accounts for stability of *g* from age to age, some change is genetically driven. For example, from 7 to 10 years, age-to-age genetic correlations are 0.70–0.80 (Davis *et al.* 2007). Because some of the TEDS sample used in the present study have been tested at 9 and 10 years and is now being tested at 12 years, it will be possible to address the issue of developmental change and continuity of *g*. However, given the increased role of heritability (and decrease of shared environment) in *g* with age, caution should be exercised when interpreting the replication of these SNP associations at other (particularly much older) ages.
6. Finally, our SNP-MaP screening was confined to the autosomes. An analysis of Online Mendelian Inheritance in Man (OMIM, http://www.ncbi.nlm.nih.gov/omim) shows that over 10% of searches for ‘mental retardation’ involved X-linked loci (Inlow & Restifo 2004), suggesting that a stratified pooled DNA approach may show novel loci on the X chromosome that contributes to *g*.

## Supplementary material

The following supplementary material is available for this article online from http://www.blackwell-synergy.com/doi/full/10.1111/j.1601-183X.2007.00368.x

Appendix S1 Derivation of pooled allele frequency estimates, composite score creation, and Table S1.
