# Supplementary material for: Genome-wide quantitative trait locus association scan of general cognitive ability using pooled DNA and 500K single nucleotide polymorphism microarrays
Source: Genes Brain Behav. 2008 Jun;7(4):435–46. doi: 10.1111/j.1601-183X.2007.00368.x (PMC2408663; doi:10.1111/j.1601-183X.2007.00368.x)
Supplement: Appendix S1 — Derivation of pooled allele frequency estimates, composite score creation, and Table S1. [file gbb0007-0435-SD1.doc]

**Supplementary Materials and Methods**

The following sections give an overview of how we calculated allele frequency estimates using the measurement structure of Affymetrix DNA genotyping microarrays with pooled DNA of groups selected for low *g* and high *g*. We also detail how these measurements (and their derivatives) were implemented to form a five-criterion rank-based composite score for each SNP used to select SNPs from Stage 1 (pooled DNA using Affymetrix 500K microarrays) for individual genotyping on an independent and representative sample of *g*.

*Calculation of allele frequency estimates using pooled DNA:*

As with other applications of pooled DNA, allele frequency estimates (*p*) are calculated as the proportion of fluorescent intensity corresponding to allele A to the fluorescent intensities corresponding to the sum of alleles *A* and *B*:

$p=\frac{A}{A+B}$(1)

Affymetrix microarrays, however, measure alleles *A* and *B* numerous times using multiple unique probes (oligos) scattered across the microarray’s surface. The fluorescent intensity values for these probes are contained in cell files (.cel) that are produced separately for each microarray. The lowest level of at which an allele frequency estimate can be measured using equation (1) is at the “quartet” level. A quartet contains four 25bp probes with variations at a consistent location within each probe. The four variations are either 1) a perfect match to allele A of the SNP (*PM^A^*), 2) a perfect match to allele B (*PM^B^*), 3) a mismatch to allele A (*MM^A^*) or 4) a mismatch to allele B (*MM^B^*). Numerous quartets represent each SNP on the microarray with variation achieved through “off-sets” and/or by designing quartets on sense or anti-sense strands. Off-sets refer to the shifting of the SNP interrogation site to a different position within the quartets’ probes. Sequence design occurs either exclusively on the sense or anti-sense strand, or on both strands depending on the SNP. Therefore, microarray measurement is defined as taking place at the *i^th^* quartet of the *j^th^* replicate in group *k*, for SNP *s*.

We denote the four probes within a quartet as:

Perfect match allele A = $PM_{ijks}^{A}$

Perfect match allele B = $PM_{ijks}^{B}$

Mismatch allele A = $MM_{ijks}^{A}$

Mismatch allele B = $MM_{ijks}^{B}$

We then transform *PM_ijks_* probe intensities by subtracting an estimate of non-specific hybridization (the average intensity of the two mismatch probes) to derive best estimates for allele A (*A_ikjs_*) and allele B (*B_ikjs_*):

$A_{ijks}=MAX\left(PM_{ijks}^{A}-\left\{\frac{MM_{ikjs}^{A}+MM_{ijks}^{B}}{2}\right\},0\right)$(2)

$B_{ijks}=MAX\left(PM_{ijks}^{B}-\left\{\frac{MM_{ikjs}^{A}+MM_{ijks}^{B}}{2}\right\},0\right)$(3)

Transformed values are then substituted into equation (1) to provide an allele frequency estimate for the *i*^th^ quartet:

$p_{ijks}=\frac{A_{ijks}}{A_{ijks}+B_{ijks}}$(4)

The allele frequency estimate for the *j*^th^ replicate is the simple arithmetic mean of *I* quartets with ‘interpretable data’, i.e., the denominator of equation (4) ≠ 0:

$p_{jks}=\left\{\begin{matrix} \frac{1}{I}\sum_{i=1}^{I} p_{ijks}, if I\geq0.7I_{\max} \\ NULL, if I<0.7I_{\max} \end{matrix}\right.$(5)

(Where $I_{\max}\in\left\{6,10\right\}$ and is dependent on SNP, *s*. As can bee seen from equation (5) we only accepted allele frequency estimates from replicates with either 5/6 or 7/10 quartets with interpretable data.)

Variance across quartets is thus:

$p_{jks}^{\mathrm{var}}=\frac{1}{I-1}\sum_{i=1}^{I} (p_{ijks}-p_{jks})^{2}$(6)

The allele frequency estimate for the *k*^th^ group is simply the arithmetic mean of *J* replicates, thus:

$p_{ks}=\frac{1}{J}\sum_{j=1}^{J} p_{jks}$(7),

with variance of allele frequency estimates across replicates:

$p_{ks}^{\mathrm{var}}=\frac{1}{J-1}\sum_{j=1}^{J} (p_{jks}-p_{ks})^{2}$(8),

where *J* is the number of replicates within the *k*^th^ group.

The following sections detail how the data acquired from the above equations is used to create a rank-based composite score (based on five criteria) for each SNP used to select SNPs from Stage 1 (pooled DNA using Affymetrix 500K microarrays) for individual genotyping on an independent and representative sample of *g*.

**Criterion (1)**

*Allele frequency difference between low and high* g *groups*

Using equation (7) we calculated allele frequency estimates separately for the two groups, low *g* ($k^{low}$) and high *g* ($k^{high}$)*.* The allele frequency difference between groups at SNP *s* is the absolute difference between group estimates, thus:

$p_{s}^{diff}=\left|p_{k^{low}s}-p_{k^{high}s}\right|$(9)

For the composite, allele frequency differences were standardized separately by array type (NspI or StyI) and weighted positively (to prioritize larger allele frequency differences) which we denote:

$$+Zp_{s}^{diff}$$

**Criterion (2)**

*Variance of allele frequency estimates across replicates*

Equation (8) was applied to each group, averaged, standardized separately by array type, summed then weighted negatively (to prioritize low variance scores), which we denote:

$$-\frac{1}{K}\sum_{k=1}^{K} Zp_{ks}^{\mathrm{var}}$$

**Criterion (3)**

*Variance of allele frequency estimates across quartets*

Equation (6) was applied to each replicate, averaged across all replicates, standardized separately by array type, summed then weighted negatively (to prioritize low variance scores), which we denote:

$$-\frac{1}{J}\sum_{j=1}^{J} Zp_{jks}^{\mathrm{var}}$$

**Criterion (4)**

*Number of replicates*

We denote the number of replicates at SNP *s*, for low *g* and high *g* groups at SNP *s* as$J_{k^{low}s}$and$J_{k^{high}s}$, respectively. We took the arithmetic mean of these values, standardized the result separately by array type, summed them then weighted them positively (to prioritize SNPs with more replicates) to give:

$$+\frac{1}{K}\left(ZJ_{k^{low}s}+ZJ_{k^{high}s}\right)$$

**Criterion (5)**

*Minor allele frequency*

Minor allele frequency was calculated as:

EMBED Equation.3

For the composite, minor allele frequencies were standardized separately by array type and positively weighted (to prioritize common allele frequencies) which we denote:

$$+Zp_{s}^{\min}$$

**Composite**

The composite measure (*C*) was the simple summation of the standardized information from criterion 1-5:

$$C=+Zp_{s}^{diff}-\frac{1}{K}\sum_{k=1}^{K} Zp_{ks}^{\mathrm{var}}-\frac{1}{J}\sum_{j=1}^{J} Zp_{jks}^{\mathrm{var}}+\frac{1}{K}\left(ZJ_{k^{low}s}+ZJ_{k^{high}s}\right)+Zp_{s}^{\min}$$

In future experiments, different composite scores may be created by assigning weights to the different criteria, or add or remove different criteria. At the time of writing, the criteria used were believed to be the most informative for detecting QTLs of small effect size.

Online-only tables

| **SNP information** | | | **Stage 1 (SNP-MaP)** | | | | | **Stage 2 (Individual genotyping)** | | | | | |  |
| --- | --- | --- | --- | --- | --- | --- | --- | --- | --- | --- | --- | --- | --- | --- |
| dbSNP rs# | Chr | genomic relation | Strand | Alleles | low *g*  (allele freq) | high *g*  (allele freq) | between-group | Strand | *r* | *p* | *N* | correct direction | HWE (*p*) | |
|  |  |  |  |  |  |  | allele freq difference |  |  |  |  |  |  |  |
| rs10513536 | 3q25.33 | mRNA evidence | + | C/G | 0.467 | 0.528 | -0.061 | - | 0.011 | 0.278 | 2667 | YES | 0.974 | |
| rs10888515 | 1q21.3 | intergenic | + | C/G | 0.528 | 0.416 | 0.112 | + | -0.031 | 0.053 | 2674 | NO | 0.068 | |
| rs10922043 | 1q31.3 | intergenic | + | C/G | 0.591 | 0.517 | 0.074 | + | -0.001 | 0.478 | 2625 | -- | 0.630 | |
| rs11181185 | 12q12 | intergenic | + | A/G | 0.535 | 0.396 | 0.139 | + | 0.001 | 0.474 | 2673 | -- | 0.650 | |
| rs11691504 | 2q31.3 | intergenic | + | A/C | 0.418 | 0.497 | -0.079 | + | 0.042 | 0.016 | 2680 | NO | 0.504 | |
| rs1173419‡ | 6q14.3 | intergenic | - | A/G | 0.449 | 0.523 | -0.074 | - | -- | -- | -- | -- | -- | |
| rs11761076 | 7q32.1 | intergenic | + | A/G | 0.374 | 0.512 | -0.138 | + | -0.045 | 0.010 | 2644 | YES* | 0.496 | |
| rs12326771 | 18q12.2 | FHOD3, intron 3 | - | A/C | 0.149 | 0.367 | -0.218 | + | -0.030 | 0.063 | 2659 | NO | 0.633 | |
| rs12982449 | 19q13.31 | mRNA evidence | - | C/G | 0.514 | 0.402 | 0.112 | + | -0.026 | 0.086 | 2680 | YES | 0.127 | |
| rs1378810 | 3q22.1 | DNAJC13, intron 55 | + | A/T | 0.478 | 0.605 | -0.127 | - | 0.062 | 0.001 | 2667 | YES* | 0.248 | |
| rs1400851‡ | 5p13.2 | WDR, intron 9 | - | A/C | 0.461 | 0.546 | -0.085 | + | -- | -- | -- | -- | -- | |
| rs1538283 | 6q16.1 | intergenic | - | C/T | 0.478 | 0.545 | -0.067 | + | -0.003 | 0.441 | 2689 | -- | 0.526 | |
| rs174455 | 11q12.3 | FADS3, intron 1 | + | A/G | 0.559 | 0.451 | 0.108 | + | 0.043 | 0.013 | 2690 | YES* | 0.297 | |
| rs1871453 | 10q22.1 | intergenic | - | A/G | 0.394 | 0.487 | -0.093 | + | -0.002 | 0.456 | 2664 | -- | 0.573 | |
| rs2006818 | 12q12 | intergenic | - | G/T | 0.449 | 0.528 | -0.079 | - | -0.016 | 0.201 | 2697 | YES | 0.940 | |
| rs2010794 | 11p15.4 | HBG2 | - | A/T | 0.505 | 0.600 | -0.095 | - | 0.003 | 0.441 | 2680 | -- | 0.802 | |
| rs2164787 | 13q22.1 | intergenic | + | G/T | 0.607 | 0.424 | 0.183 | + | 0.010 | 0.302 | 2661 | YES | 0.989 | |
| rs2167905 | 11p13 | intergenic | - | C/T | 0.552 | 0.455 | 0.097 | - | 0.015 | 0.221 | 2675 | YES | 0.730 | |
| rs2243057‡ | 5q13.3 | F2RL1, intron 1 | - | C/T | 0.553 | 0.709 | -0.156 | + | -- | -- | -- | -- | -- | |
| rs2253020 | 7q11.23 | HIP1, intron 1 | + | A/G | 0.344 | 0.485 | -0.141 | + | -0.017 | 0.194 | 2691 | YES | 0.139 | |
| rs2381536 | 9p24.1 | JMJD2C, intron 15 | - | C/T | 0.471 | 0.562 | -0.091 | + | 0.001 | 0.470 | 2642 | -- | 0.817 | |
| rs2402007 | 7q31.2 | TFEC, intron 5 | + | A/T | 0.553 | 0.681 | -0.128 | + | -0.027 | 0.077 | 2686 | YES | 0.655 | |
| rs2496143 | 6p24.1 | TBC1D7, intron 5 | - | A/G | 0.52 | 0.453 | 0.067 | + | -0.034 | 0.037 | 2704 | YES* | 0.238 | |
| rs251316 | 5q12.3 | intergenic | + | A/C | 0.564 | 0.489 | 0.075 | + | -0.008 | 0.335 | 2648 | -- | 0.197 | |
| rs2867327 | 5q31.1 | mRNA evidence | + | A/G | 0.55 | 0.461 | 0.089 | + | 0.018 | 0.178 | 2679 | YES | 0.864 | |
| rs3787569 | 20p13 | ANGPT4, intron 1 | + | A/G | 0.813 | 0.644 | 0.169 | + | -0.020 | 0.152 | 2648 | NO | 0.071 | |
| rs394623‡ | 16q24.1 | C16orf74, intron 1 | - | C/G | 0.598 | 0.508 | 0.09 | - | -- | -- | -- | -- | -- | |
| rs4571814 | 9p21.3 | MLLT3, intron 2 | + | G/T | 0.637 | 0.381 | 0.256 | + | 0.003 | 0.432 | 2661 | -- | 0.477 | |
| rs4664767 | 2q24.1 | intergenic | + | C/T | 0.437 | 0.534 | -0.097 | + | 0.012 | 0.263 | 2665 | NO | 0.002 | |
| rs4818094† | 21q22.2 | LOC150084, intron 5 | - | A/G | 0.408 | 0.281 | 0.127 |  | -- | -- | -- | -- | -- | |
| rs4936168 | 11q25 | OPCML, intron 4 | - | A/G | 0.547 | 0.435 | 0.112 | + | -0.010 | 0.303 | 2650 | YES | 0.443 | |
| rs629922‡ | 11q23.2 | ZBTB16, intron 4 | + | C/T | 0.458 | 0.536 | -0.078 | + | -- | -- | -- | -- | -- | |
| rs6436196 | 2q35 | intergenic | + | C/T | 0.545 | 0.435 | 0.11 | + | 0.003 | 0.428 | 2687 | -- | 0.993 | |
| rs6584579 | 10q24.33 | OBFC1, intron 9 | + | A/G | 0.586 | 0.505 | 0.081 | + | -0.019 | 0.158 | 2661 | NO | 0.766 | |
| rs6790570 | 3p14.1 | SUCLG2, intron 1 | - | A/G | 0.503 | 0.406 | 0.097 | + | 0.011 | 0.280 | 2671 | NO | 0.669 | |
| rs6794128† | 3p13 | PDZRN3, intron 3 | - | C/T | 0.587 | 0.502 | 0.085 |  | -- | -- | -- | -- | -- | |
| rs6904862† | 6p22.3 | intergenic | - | A/G | 0.638 | 0.551 | 0.087 |  | -- | -- | -- | -- | -- | |
| rs7195954 | 16p13.3 | intergenic | - | C/G | 0.621 | 0.717 | -0.096 | + | 0.033 | 0.043 | 2655 | YES* | 0.990 | |
| rs7237066 | 18q11.2 | intergenic | + | A/C | 0.54 | 0.467 | 0.073 | + | 0.011 | 0.295 | 2634 | YES | 0.985 | |
| rs7284992 | 22q13.32 | intergenic | + | A/G | 0.49 | 0.557 | -0.067 | + | 0.006 | 0.376 | 2655 | -- | 0.734 | |
| rs759708† | 14q24.3 | C14orf149, intn. 14 | + | A/G | 0.446 | 0.566 | -0.12 |  | -- | -- | -- | -- | -- | |
| rs7920351 | 10q26.3 | intergenic | + | A/G | 0.563 | 0.488 | 0.075 | + | 0.004 | 0.421 | 2672 | -- | 0.788 | |
| rs883686 | 4q26 | PRSS12, intron 4 | + | A/G | 0.466 | 0.594 | -0.128 | - | 0.031 | 0.054 | 2689 | YES | 0.869 | |
| rs924765 | 8q24.22 | intergenic | - | C/G | 0.472 | 0.574 | -0.102 | + | -0.006 | 0.378 | 2669 | -- | 0.960 | |
| rs9513824† | 13q32.3 | mRNA evidence | + | C/T | 0.533 | 0.667 | -0.134 |  | -- | -- | -- | -- | -- | |
| rs9559583 | 13q33.3 | intergenic | - | A/G | 0.446 | 0.542 | -0.096 | + | 0.011 | 0.290 | 2677 | YES | 0.867 | |
| rs959922 | 5q15 | intergenic | - | C/T | 0.559 | 0.491 | 0.068 | - | 0.024 | 0.106 | 2664 | YES | 0.047 | |

*Table S1*: Summary of Stage 1 and Stage 2 genotyping for the 47 SNPs selected by ranked composite score using pooled DNA to screen the genome. These SNPs were individually genotyped on an independent sample of low and high *g* individuals to confirm selection. The allele frequency difference denoted in the 8^th^ column from the left is calculated by subtracting the allele frequency of the High *g* group from the Low *g* group. The allele measured in the ‘Low *g*’ and ‘High *g*’ columns is the allele which is alphabetically first in the ‘Alleles’ column (e.g., C rather than G in the first row). Note that the alleles measured here (using Affymetrix microarrays) may not be the same as the alleles measured using individual genotyping (using the SNPlex™ assay) because the Affymetrix and SNPlex platforms may be based on different DNA strands. The ‘correct direction’ column indicates whether the direction of allele frequency differences is the same in Stage 2 as it was in Stage 1. We did not compare directions of effect between Stage 1 and Stage 2 for SNPs showing correlations in the range -.01 and .01 in Stage 2.

† Assay failure: no data available

‡ Poor genotype concordance (< .95) with HapMap: SNP omitted

* SNP significantly associated across full range of *g* scores: SNP selected for inclusion in SNP set.
